# Transfer of Communication Skills to the Workplace during Clinical Rounds: Impact of a Program for Residents

**DOI:** 10.1371/journal.pone.0012426

**Published:** 2010-08-26

**Authors:** Aurore Liénard, Isabelle Merckaert, Yves Libert, Isabelle Bragard, Nicole Delvaux, Anne-Marie Etienne, Serge Marchal, Julie Meunier, Christine Reynaert, Jean-Louis Slachmuylder, Darius Razavi

**Affiliations:** 1 Institut Jules Bordet, Brussels, Belgium; 2 Faculté des Sciences Psychologiques et de l'Éducation, Université Libre de Bruxelles, Brussels, Belgium; 3 Faculté de Psychologie, Université de Liège, Liège, Belgium; 4 Hôpital Erasme, Brussels, Belgium; 5 C.P.O. (Centre de Psycho-Oncologie), Brussels, Belgium; 6 Faculté de Psychologie et des Sciences de l'Éducation, Université Catholique de Louvain, Louvain-la-Neuve, Belgium; Women's and Children's Hospital, Australia

## Abstract

**Background and Purpose:**

Communication with patients is a core clinical skill in medicine that can be acquired through communication skills training. Meanwhile, the importance of transfer of communication skills to the workplace has not been sufficiently studied. This study aims to assess the efficacy of a 40-hour training program designed to improve patients' satisfaction and residents' communication skills during their daily clinical rounds.

**Methods:**

Residents were randomly assigned to the training program or to a waiting list. Patients' satisfaction was assessed with a visual analog scale after each visit. Transfer of residents' communication skills was assessed in audiotaped actual inpatient visits during a half-day clinical round. Transcripted audiotapes were analyzed using content analysis software (LaComm). Training effects were tested with Mann-Whitney tests and generalized linear Poisson regression models.

**Results:**

Eighty-eight residents were included. First, patients interacting with trained residents reported a higher satisfaction with residents' communication (Median = 92) compared to patients interacting with untrained residents (Median = 88) (p = .046). Second, trained residents used more assessment utterances (Relative Risk (RR)  = 1.17; 95% Confidence intervals (95%CI)  = 1.02–1.34; p = .023). Third, transfer was also observed when residents' training attendance was considered: residents' use of assessment utterances (RR = 1.01; 95%CI = 1.01–1.02; p = .018) and supportive utterances (RR = 0.99; 95%CI = 0.98–1.00; p = .042) (respectively 1.15 (RR), 1.08–1.23 (95%CI), p<.001 for empathy and 0.95 (RR), 0.92–0.99 (95%CI), p = .012 for reassurance) was proportional to the number of hours of training attendance.

**Conclusion:**

The training program improved patients' satisfaction and allowed the transfer of residents' communication skills learning to the workplace. Transfer was directly related to training attendance but remained limited. Future studies should therefore focus on the improvement of the efficacy of communication skills training in order to ensure a more important training effect size on transfer.

## Introduction

Communication skills are recognized as one of physicians' core clinical skills. Effective communication skills are the key to achieve the three main purposes of physician–patient relationship: assessment, support and information [1]. Effective assessment, support and information may improve patients' satisfaction [2] and psychological adjustment [3]. A few studies have shown that these skills may be learned and transferred to physicians' clinical practice after a communication skills training program [4]–[7]. Transfer of learned skills to clinical practice has been shown to remain limited however. The importance of transfer of communication skills training to the workplace should thus be studied further.

In theory, communication skills should be acquired by physicians as early as possible, that is during undergraduate training or residency. Although communication skills training are increasingly organised for undergraduates, residency remains an appropriate period, not only to learn communication skills but also, to transfer learned communication skills to the clinical practice as residents' daily practice becomes more varied and challenging (clinical rounds and/or outpatients consultations). According to the Baldwin and Ford model [8], transfer depends directly on learning, and is influenced by trainees' characteristics, work environment and training program content and duration. Several communication skills training programs have been organised for residents [9]–[16]. Among these programs, only three controlled studies have shown efficacy in terms of transfer of learned skills to clinical practice [9], [11], [12]: only two of these studies had been randomized [9], [11]. It should be noted that the way transfer has been assessed in these studies is not optimal - only one consultation with an actual patient assessed [9], and only one study assessing patients' outcomes such as patients' satisfaction [11].

Transfer of skills by residents after a communication skills training has moreover never been assessed during clinical rounds which is an important part of residents' clinical practice. During clinical rounds, residents have short and frequent visits with inpatients and the purposes of these visits are numerous (assessment, information, support and treatment management). There is thus still a need to develop randomized controlled studies designed to assess the efficacy of this type of training in terms of transfer of learned skills to clinical practice.

The aim of this study was to assess in a randomized controlled design the impact of a communication skills training program (The Belgian Interuniversity Curriculum - Communication Skills Training (BIC-CST)) [17] on the transfer by residents of learned skills during a half-day clinical round. Transfer was measured through the assessment of patients' satisfaction with residents' communication and through the assessment of residents' communication skills in a half-day of clinical round. First, it was hypothesized that a communication skills training would lead to a higher level of patients' satisfaction with residents' communication skills used during the visits. Second, it was hypothesized that a communication skills training would lead to an increase in residents' use of assessment and supportive skills.

## Methods

### Ethics statement

The ethics committee of Jules Bordet Institute (Brussels) approved of the study. Residents and patients included in the study had to give their written informed consent.

### Subjects

To be included in this study, residents had to speak French and to be willing to participate in the training program and its assessment procedure. Residents had also to have worked, be working with, or be in a project working with, cancer patients (part or full time). Residents participating in another psychological training program during the assessment and training periods were excluded from the study.

### Study design and assessment procedure

**Figure 1:**
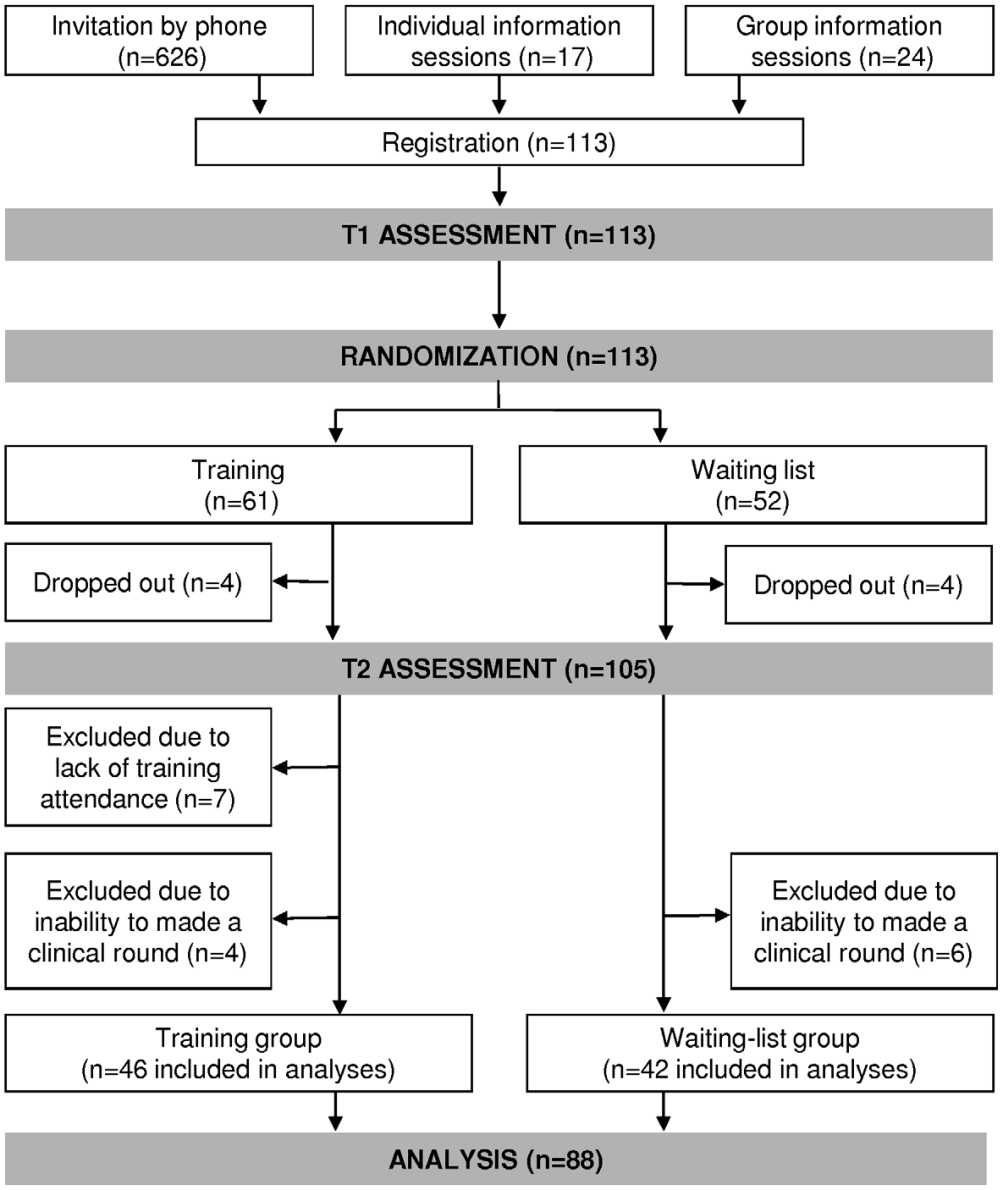
Recruitment procedure, study design, training and assessment procedures. T1: assessments scheduled before the training program; T2: assessments 8 months after the first assessment.

The efficacy of the Belgian Interuniversity Curriculum - Communication Skills Training (BIC-CST) was assessed in a study allocating residents after the first assessment time to a 40-h training program (training-group) or to a waiting list (waiting-list-group), according to a computer generated randomization list. As displayed in Figure 1, assessments were scheduled before randomization (T1) and after the training program for the training-group (T2) and 8 months after T1 for the waiting-list-group (T2). At each assessment time, the procedure included, among other, visits with actual patient during a half-day clinical round.

### Training Program

The Belgian Interuniversity Curriculum - Communication Skills Training (BIC-CST) is a 40-hour training program designed for residents which includes a 30-h communication skills training module and a 10-h stress management module [17]. Sessions were spread bimonthly over an 8-month period. The program was organized in small groups (up to 7 participants). BIC-CST was learner-centered, skills-focused, practice-oriented and tailored to residents' needs. It included a cognitive, a behavioral and a modeling component.

The communication skills training module consisted of a 17-h communication skills training focusing on two-person consultations, a 10-h communication skills training program focusing on three-person consultations (that is where a relative accompanies the patient) and lastly a 3-h session promoting integration and use of learned skills (communication and stress management). Among the 30 hours devoted to communication skills learning, a 1-h session focused on theoretical information. In the other sessions, residents were invited to practice communication skills through predefined role plays (on breaking bad news) and through role plays based on the clinical problems arising from their everyday clinical round practice (e.g. breaking bad news, end-of-life discussion, interaction with patient facing complex medical situations, patients' emotional reaction, …). Residents were given immediate feedback on the communication skills performed during role plays.

BIC-CST was specifically designed to focus on transfer of learned skills to clinical rounds. First, training sessions were scheduled bimonthly in order to allow residents sufficient time to transfer learned communication skills to their clinical practice. Second, role plays based on clinical problems brought up by the residents were scheduled to allow them to test the usefulness of learned communication skills and to facilitate their transfer to the clinical situations perceived as problematic. Third, trainers' feedback was adjusted to each resident's skill level in order to increase their self-efficacy about transfer. Finally, trainers were asked to support and encourage residents' transfer of learned communication skills to their clinical practice. At the beginning of each session they debriefed residents about their use of learned skills and encouraged them to pursue their efforts and at the end of each session they encouraged residents to test learned skills in their clinical practice. The choice of the skills taught was based on results of studies that have shown the positive impact of using specific patient-centered communication skills (such as open and open-ended question and empathy) [18].

Residents' attendance to the 30-hour communication skills module has been considered to analyze the training attendance effect on transfer of communication skills to clinical practice.

### Actual patient visits

Transfer was assessed in actual inpatient visits during a half-day clinical round at the two assessment times. During this half-day, all residents' visits with included patient were audiotaped. To be included, patients had to be more than 18 years old, able to speak French, free of any cognitive dysfunction, alone during the visit, exempt of any medical contraindication. Different patients were studied at the two timepoints, T1 and T2. During the half-day assessment, a set of questionnaires was completed by residents and also by patients.

### Communication Content Analysis

The audiotapes of the actual patient visits were transcribed and the transcripts analyzed by the LaComm software. LaComm is a French communication content analysis software. This software uses a word count strategy based on categories of words like Protan [19] or Linguistic Inquiry Word Count [20] and a word combination strategy like the General Inquirer [21]. The aim of this software is to analyze, utterance by utterance, verbal communication used (in medicine in general and in oncology in particular) by identifying utterance types and contents.

Regarding utterance types, communication used during consultations was analyzed with the dictionaries included in the LaComm. Dictionaries are composed of words, word stems or expressions and were built on the basis of empirical knowledge derived from actual and simulated patient consultations performed by physicians [7], [22]. The categories of dictionaries were adapted from the categories of the Cancer Research Campaign Workshop Evaluation Manual [7], [22], [23], [24] and redefined according to the three-function approach of the medical consultation [1] by a panel of experts (Table 1). Utterances were categorized in three main types: assessment; support; and information. Regarding utterance contents, three dictionaries were constructed: medical, emotional, and social.

**Table 1:** Description of the utterance types and contents provided by the Lacomm (communication content analysis software).

	Definitions	Examples
**Utterance types**		
*Assessment*		
Open questions	Assessment of a wide range of issues, concerns, or feelings.	How are you doing? ; Tell me.
Open directive questions	More focused assessment of issues, concerns, or feelings.	Tell me what occured since the last treatment. ; What do you feel about it?
Directive questions	Precise assessment of a specific area.	Did you begin the treatment? ; Are you feeling pain?
Leading questions	Assessment of a more precise dimension while suggesting an answer.	You do not have pain, don't you?
Checking questions	Checking of information given without seeking further elaboration.	Really? ; Do you understand what I say?
Other types of questions	Assessments not classified by LaComm into one of the previous categories.	
*Support*		
Acknowledgement	Support by listening to the patient.	Mh, Mh. ; Right. ; That should not be easy.
Empathy	Support by showing an understanding of the patient's emotional or physical state.	I understand that you are distressed. ; I realize that you have severe pain.
Reassurance	Support by reassuring the patient about a potential threat, discomfort or uncertainty.	Don't worry. ; I will do everything that is possible to help you.
*Information*		
Procedural information	Information about orientation and transition of talk in the consultation.	I am Doctor x. ; Please take a seat.
Negociation	Proposition to the patient taking his/her point of view into account.	I suggest we talk about it with your husband.
Other types of information	Affirmative utterrances not classified by LaComm into one of the previous categories.	
**Utterance contents**		
*Medical words*	Words related to oncology and other medical specialities such as diagnosis, prognosis, techniques, biological terms, …	Cancer, lesions, palliation, chemotherapy, blood, breast, exams, pain.
*Emotional words*	Words related to negative and positive emotion.	Fear, sad, happy, anxious, confort, suffering, satisfaction.
*Social words*	Words related to relation and daily life (hobbies, clothes, food,…).	Partner, work, hobby, driving, children, shopping.

The content analysis software has been shown to be effective in measuring improved communication skills [25], [26]. It allows analyses of verbal communications which reflect important aspects of medical interactions. It is important to underline that this software is only useful to assess training effects and is not designed for teaching.

### Questionnaires

#### Patients' satisfaction with residents' communication skills

**Figure 2:**
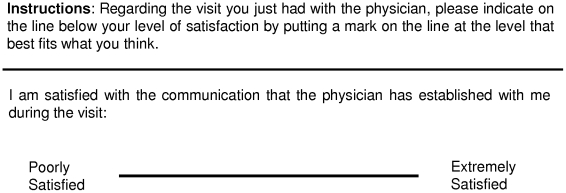
Patients' satisfaction with residents' communication skills recorded through a visual analogue scale.

This 3-item questionnaire assesses patients' satisfaction with the medical visit. One of these items consisted of a measure of patients' satisfaction with residents' communication skills used during the medical visit. After the clinical round visits, patients rated their satisfaction level on a 10 cm visual analogue scale (VAS). A VAS was chosen here to provide a more sensitive measure of patients' satisfaction with residents' communication as ratings are not restricted to response categories [27]. Because patients often report a high level of satisfaction [28], ratings responses ranged from “poorly satisfied” (0) to “extremely satisfied” (10) (Figure 2). This unbalanced response option with more positive than negative levels was chosen to provide a more sensitive measure as such response options are more likely to spread out favorable opinions and thus provide a less positively skewed score distribution [29].

### Statistical Analyses

To be considered for data analysis, residents had to attend at least one hour of communication skills training. To analyse patients' satisfaction, all visits of a half-day clinical round were considered at each assessment time. A mean score of patients' satisfaction was calculated for each resident per half-day clinical round. Differences between the training group and waiting-list group were assessed at baseline and at the second assessment time using non parametric tests for independent groups such as, the Mann-Whitney test.

To analyse the Lacomm data, one visit was selected by resident at the second assessment time according to visit duration. For each resident, the visit was selected so that its duration was the closest to the median duration of all visits. This choice was made because visit duration is a covariate of the number of communication skills used. Patient characteristics and visit characteristics at the second assessment time were compared using Student's t test and X^2^ tests as appropriate. Data generated from the LaComm are in counts of utterance types and contents. The LaComm data considered as the dependent variables were tested with generalized linear equation Poisson regression models according to two models: the one assessed training effects (group allocation) and the other assessed residents' training attendance effects (number of hours of attendance to the communication skills module (30 hours maximum)) using the waiting-list group as the reference group. These two models have been adjusted for the number of residents' turns of speech. All tests were two-tailed, and the alpha was set at 0.05. Analyses were performed with SPSS Version 16.0 for PC (SPSS Inc, Chicago, IL).

## Results

### Resident Recruitment and Sociodemographic Data

One-hundred and thirteen residents registered to the Belgian Interuniversity Curriculum - Communication Skills Training (BIC-CST) (Figure 1). Eighty-eight residents were considered for analyses. Concerning residents' sociodemographic and socioprofessional characteristics, no statistically significant differences were found at baseline between trained residents (training-group residents) and untrained residents (waiting-list-group residents).

Trained residents were a mean of 28 years old (SD = 3 years), 67% were female, 39% lived alone. Nine residents (20%) were in their first year of residency, 12 in the second year (26%), 15 in the third year (32%), 5 in the fourth year (11%) and 5 in the fifth year (11%). Seven percent were residents in oncology (oncology, haematology and radiotherapy), 30% in gynaecology and 63% in other specialities (surgery, gastroenterology …). Five residents had attended a brief communication skills training workshop in the last year. Untrained residents were a mean of 28 years old (SD = 1.5 years), 62% were female, 24% lived alone. Seven residents (17%) were in their first year of residency, 4 in the second year (9%), 16 in the third year (38%), 10 in the fourth year (24%) and 5 in the fifth year (12%). Twenty-one percent were residents in oncology, 21% in gynaecology and 58% in other specialities. No resident had attended a brief communication skills training workshop in the last year.

Trained residents took part on average, 25 hours of the training program (SD = 8.3; Min = 8; Max = 40). They participated on average, 8 hours of the stress management skills module (SD = 2.4; Min = 3.5; Max = 10) and 17 hours of the communication skills module (SD = 6.8; Min = 3; Max = 30).

### Patient recruitment Data

Concerning the recruitment procedure, 1260 patients were met by the assessable residents during the half-day clinical rounds (686 at baseline and 574 at the second assessment time). Three hundred and seventy-seven patients (30%) were ineligible for one or more reasons: Eight patients were younger than 18 years old, 75 were not fluent enough in French to complete the study, 86 presented cognitive dysfunctions, 135 were excluded for medical reasons and 79 were accompanied by a relative. One hundred and twenty-six (10%) refused to participate: 15 patients refused because of time constraints; 20 for intimacy reasons; 16 refused because of visits recording; 46 felt that they were not fit enough to complete the questionnaire and 57 refused for another reason. Seven hundred and fifty-seven (60%) were thus included in the study (390 at baseline and 367 at the second assessment time). Each resident had on average 4.5 clinical round visits (SD = 2; Min = 1; Max = 13) per half-day of assessment included in this study.

### Training effects on patients' satisfaction

Four residents, three in the training-group and one in the waiting-list-group, who did not have a clinical round during the first assessment time, were not included in this analysis. For this analysis, 390 patients were included at baseline (206 for the trained group and 184 for the waiting-list group) and 341 at the second assessment (182 for the trained group and 159 for the waiting-list group). It should be recalled that a mean satisfaction level was computed including for each resident all visits of their half-day clinical round. No group differences were observed regarding clinical round visits characteristics at baseline and at the second assessment time.

Regarding patients' satisfaction, Mann-Whitney tests did not show significant differences between groups at baseline (p = .366) but showed significant differences between groups at the second assessment time (p = .047) (Table 2). Patients' satisfaction levels were higher in the training group (Median = 92; Q1–Q3 = 87–97) compared to the waiting-list group (Median = 88, Q1–Q3 = 83–95).

**Table 2:** Training effects on characteristics of half-day clinical round visits (mean visits duration and mean number of turns of speech by visits) and on patients' satisfaction (mean patients' satisfaction by half-day) (n = 84).

	T1						T2					
	TG		WLG		Mann-Whitney		TG		WLG		Mann-Whitney	
	Med	Q1–Q3	Med	Q1–Q3	*z*	*p*	Med	Q1–Q3	Med	Q1–Q3	*z*	*p*
**Visits duration** *	7.0	5.0–10.7	8.2	4.6–10.7	−.25	.802	6.4	5.0–10.0	7.0	5.4–9.9	−.18	.854
**Turns of speech**												
Residents	68	50–96	64	49–92	−.59	.558	67	52–95	72	47–93	−.32	.751
Patients	66	49–95	63	48–91	−.53	.598	67	51–92	71	47–90	−.33	.741
**Patients**' **satisfaction**	88	81–93	89	84–93	−.90	.366	92	87–97	88	83–95	−1.99	.046

*Visits duration are expressed in minutes. T1: at baseline; T2: after training for the training group and after 8 months for the waiting-list group; TG: Training Group (n = 43); WLG Waiting-List Group (n = 41); Med: Median.

### Training effects on residents' and patients' utterances

For the utterances analysis, one visit was selected by resident at the second assessment time according to visit duration. Patients' sociodemographic, disease and visits characteristics were reported in table 3. There was no statistically significant difference between patients met by trained and by untrained residents.

**Table 3:** Characteristics of selected visit (one by resident) and patients met by resident: training and waiting-list comparison (n = 88). *

	Training group			Waiting-list group		
	(n = 46)			(n = 42)		
	n		%	n		%
**Visits characteristics**						
*Duration (minutes)*						
Mean		7.1			6.2	
SD		3.4			1.8	
*Type of physician-patient relationship*						
First encounter	11		23.9	9		21.4
Seen previously	35		76.1	33		78.6
*Type of news*						
Bad	9		19.6	5		11.9
Neutral and/or good	37		80.4	37		88.1
**Patients' sociodemographic characteristics**						
*Age*						
Mean		57.6			54.6	
SD		19.4			19.9	
*Gender*						
Male	21		45.7	18		42.9
Female	25		54.3	24		57.1
*Living with partner*						
Yes	25		54.3	20		47.6
No	21		45.7	22		52.4
*Children*						
Yes	39		84.8	32		76.2
No	7		15.2	10		23.8
*Occupational status*						
Working part or full time	7		15.2	5		12.0
Invalid, incapacitated	4		8.7	8		19.0
unemployed, homemaker, or retired	35		76.1	29		69.0
*Educational level*						
High school graduation or less	37		80.4	32		76.2
College or university graduation	9		19.6	10		23.8
**Patients' medical characteristics**						
*Type of disease*						
Pre and post partum conditions	6		13.0	7		16.7
Acute diseases	14		30.5	11		26.1
Cancer chronic diseases	16		34.8	17		40.5
Non cancer chronic diseases	10		21.7	7		16.7
*Prognosis* °						
Less than one year	12		26.7	10		24.4
One year or more	33		73.3	31		75.6
*Karnofsky score*						
80 or more	33		71.7	29		69.0
Less than 80	13		28.3	13		31.0

*Visit was selected on the basis of its duration (see method) after training for residents in the training group and at the second assessment for residents in the waiting-list group.

° two physicians could not give an opinion on patient's prognosis.

Note: no statistically significant differences were found between groups (Chi-square and t student).

As regards residents' utterances, generalised linear Poisson regression analysis showed no training effects on residents' utterances contents but showed significant effects on residents' utterance types (table 4). At the second assessment, Poisson regression showed a significant increase in the rate of assessment utterances (RR = 1.67; p = .027) for trained residents compared with untrained residents. As regards patients' utterances, analysis showed no training effect (table 4).

**Table 4:** Training and training attendance effects on the content of a selected resident visit (number of types and contents of residents' utterances and contents of patients' utterances) (n = 88).

	Training Group		Waiting-list Group		Generalised linear Poisson regression models°									
					Training effects					Training attendance effects				
					(Training vs Waiting-List)					(per hour)				
	Mean	SD	Mean	SD	RR	CI 95%			*p*	RR	CI 95%			*p*
**Residents' utterances**														
***Types***														
*Assessment*														
Open questions	0.8	0.7	0.7	0.7	1.17	0.79	to	1.72	.433	1.01	0.99	to	1.04	.192
Open directive questions*	1.8	2.0	1.2	1.4	1.27	0.85	to	1.89	.254	1.02	0.99	to	1.04	.192
Directive questions	6.8	4.9	5.9	3.5	1.10	0.85	to	1.43	.464	1.00	0.99	to	1.01	.685
Leading questions*	0.0	0.0	0.1	0.2	-	-		-	-	-	-		-	-
Checking questions	2.5	2.4	1.8	1.8	1.35	0.90	to	2.03	.146	1.02	1.00	to	1.04	.108
Other types of questions	11.1	6.5	8.9	7.4	1.17	1.02	to	1.34	.023	1.01	1.01	to	1.02	.018
Total	23.0	12.4	18.5	10.7	1.17	0.94	to	1.46	.164	1.01	0.99	to	1.02	.155
*Support*														
Acknowledgement	24.3	20.4	22.5	14.4	0.84	0.70	to	1.01	.062	0.99	0.98	to	1.00	.055
Empathy*	0.1	0.3	0.1	0.2	-	-		-	-	1.15	1.08	to	1.23	<.001
Reassurance*	0.3	0.6	0.5	0.8	0.52	0.24	to	1.12	.093	0.95	0.92	to	0.99	.012
Total	24.7	20.7	23.0	14.6	0.84	0.70	to	1.00	.053	0.99	0.98	to	1.00	.042
*Information*														
Procedural information	4.9	2.2	4.0	2.3	1.16	0.94	to	1.43	.173	1.00	0.99	to	1.01	.541
Negociation*	0.5	0.8	0.6	1.2	0.86	0.42	to	1.78	.683	0.99	0.95	to	1.02	.478
Other types of information	24.4	15.0	19.7	13.9	1.18	0.90	to	1.55	.224	1.01	0.99	to	1.02	.190
Total	29.8	15.1	24.3	14.8	1.17	0.93	to	1.47	.174	1.01	0.99	to	1.02	.192
***Contents***														
*Medical words*	21.0	12.0	17.8	10.5	1.09	0.87	to	1.38	.463	1.00	0.99	to	1.02	.587
*Emotional words* *	1.9	2.2	1.3	1.3	1.11	0.75	to	1.63	.613	1.02	0.99	to	1.03	.121
*Social words*	9.3	5.8	10.1	5.2	0.89	0.70	to	1.12	.311	1.00	0.99	to	1.01	.529
**Patients' utterances**														
***Contents***														
*Medical words*	13.4	12.9	9.9	8.6	1.15	0.81	to	1.64	.435	1.00	0.99	to	1.02	.708
*Emotional words* *	1.9	2.9	1.1	1.6	1.05	0.59	to	1.88	.870	1.00	0.98	to	1.02	.963
*Social words*	9.9	9.7	9.5	7.6	0.86	0.62	to	1.18	.341	0.99	0.97	to	1.01	.302

Note: the visit was selected on the basis of its duration (see method) after training for resident in the training group and at the second assessment for residents in the waiting-list group.

° Estimated relative rate based on a generalized linear Poisson regression models adjusted for the number of residents' turns of speech.

*Negative binomial distribution; SD. Standard deviation; RR. Relative Risk; - analyses can not be computed.

### Training attendance effects on residents' and patients' utterances

Training attendance effects test the impact of training according to the number of hours of attendance to the communication skills module (30-hour). As regards residents' utterances, generalised linear Poisson regression analysis did not show effects of residents' attendance to BIC-CST on their utterance contents but showed significant effects on residents' utterance types (table 4). At the second assessment, Poisson regression showed per hour of attendance a significant increase in the rate of the other type of assessments (RR = 1.01; p = .018) and of empathy (RR = 1.15; p<.001). Poisson regression showed also per hour of attendance a significant decrease in the rate of supportive utterances (total) (RR = 0.99; p = .042) and of reassurances (RR = 0.95; p = .012) and a marginally significant decrease in the rate of acknowledgments (RR = 0.99; p = .053).

There was no effect on residents' training attendance to BIC-CST on patients' utterances (table 4).

## Discussion

This is the first study assessing in a randomized controlled design the impact of a communication skills training program on transfer by residents of learned skills to their clinical rounds. The training program, assessed in this study, is a Belgian Interuniversity Curriculum - Communication Skills Training (BIC-CST) [17] designed specifically for residents. Transfer of learned skills has been assessed, on the one hand, by comparing satisfaction of patients interacting with trained and untrained residents and, on the other hand, by comparing trained and untrained residents' communication skills during a half-day of clinical round. It should be recalled at this level that the choice of a half-day clinical round has been made to allow the precise study of transfer of learned skills to the clinical practice. Results of this study show that trained residents successfully transferred learned skills to clinical rounds.

It was hypothesized that BIC-CST would lead to an increase in patients' satisfaction with residents' communication skills. Results showed that patients interacting with trained residents were more satisfied about residents' communication skills than patients interacting with untrained residents.

As regards residents' communication skills, it was hypothesized that BIC-CST would lead to an increase in residents' use of assessment and supportive skills during clinical rounds. Results showed that trained residents used about 20% more assessment utterances during their clinical round visits than untrained residents. Results showed meanwhile no statistically significant training effect on residents' use of supportive skills, although it should be noticed, that trained residents used about 16% less supportive utterances (marginally statistically significant effect). It should also be noticed that there is no training effect on residents' use of empathy.

Training attendance was heterogeneous. The heterogeneity is related mainly to residents' difficulty to attend training sessions due to their work overload. This heterogeneity has allowed to assess residents' training attendance - number of hours - effect on the transfer of skills. Results showed that residents' training attendance had an impact on residents' assessment and supportive utterances.

More precisely, trained residents used per hour of training one percent of assessment utterances more than untrained residents. For example, a resident attending all the 30 hours of the communication skills training module, will use 30% more assessment utterances than untrained residents. Moreover, per hour of training, changes were observed in trained residents' use of supportive utterances compared to untrained residents. It should be recalled that supportive utterances assessed in this study included acknowledgment (which refers to a simple general support), reassurance (which refers to generalisations and which are often premature in the context of clinical round visits) and empathy (which refers to a focused and explicit support). Results of this study show that trained residents, per hour of training, used one percent less acknowledgment, five percent less reassurance and fifteen percent more empathy, than untrained residents. These results show thus that residents' training attendance is directly related to the size of transfer.

The transfer found in this study may be considered as clinically relevant and is directly related to the number of hours of residents' training attendance. It should be recalled that clinical round visits in this study lasted only 8 minutes on average. In this context, an increase of one or two effective communication skills such as an assessment or an empathy is certainly clinically useful. The results reported in this study about the impact of the training program on patients' satisfaction support this idea.

Results of this study show that BIC-CST promotes the transfer by residents of learned skills to clinical practice. BIC-CST allows a more patient-centred communication during residents' clinical round visits and this patient-centred communication seems to lead patients to be more satisfied. It is important to underline that residents' participation to the communication training was heterogeneous. Results underline moreover that transfer is directly related to this level of residents' training attendance.

The study has some limitations. First, it should be recalled that residents' training attendance was heterogeneous. In view of results about the influence of training attendance on transfer, this heterogeneity may have influenced the overall level of the effect found in this study. Second, regarding training, pre-determine role-plays and clinical role-plays based on clinical problem brought up by the residents were often difficult situations rather than routine visits. Even if the practice of difficult situations may facilitate transfer [8], this choice of role-plays by residents may have had an influence on the effect size of communication skills transfer to clinical practice. Third, this study reports only the assessment of training based on a content analysis software of verbal communication. Other aspects of the resident-patient relationship (e.g. empathy based on non-verbal communication) have not been assessed.

The study of transfer of learned skills to clinical practice has become one of the most important objectives of studies assessing the efficacy of communication skills training programs. The next generation of studies designed to assess transfer should focus on the generalisation of transfer of learned skills to different specific clinical situations and on the maintenance of transfer over time. In this perspective, future studies should focus on improving the efficacy of communication skills training programs in order to ensure a more important training effect size on transfer. Studies of transfer to clinical practice should also assess, on the one hand, besides patients' satisfaction, other potential benefits for patients (patient anxiety, patient information recall, and patient compliance) and, on the other hand, besides physicians' communication skills, other physicians' outcomes (stress, burnout, self-efficacy, and satisfaction). Studies about the transfer to clinical practice should be encouraged although the cost of such initiatives is high.
